# *In vivo* magnetic resonance imaging of treatment-induced apoptosis

**DOI:** 10.1038/s41598-019-45864-y

**Published:** 2019-07-02

**Authors:** Xiaoyu Jiang, Eliot T. McKinley, Jingping Xie, Hua Li, Junzhong Xu, John C. Gore

**Affiliations:** 1 Institute of Imaging Science, Vanderbilt University, Nashville, TN 37232 USA; 2 Department of Radiology and Radiological Sciences, Vanderbilt University, Nashville, TN 37232 USA; 3 Department of Medicine, Vanderbilt University Medical Center, Nashville, TN 37232 USA; 4 Vanderbilt-Ingram Cancer Center, Vanderbilt University, Nashville, TN 37232 USA; 5 Department of Physics and Astronomy, Vanderbilt University, Nashville, TN 37232 USA; 6 Department of Biomedical Engineering, Vanderbilt University, Nashville, TN 37232 USA; 7 Department of Molecular Physiology and Biophysics, Vanderbilt University, Nashville, TN 37232 USA

**Keywords:** Cancer imaging, Magnetic resonance imaging

## Abstract

Imaging apoptosis could provide an early and specific means to monitor tumor responses to treatment. To date, despite numerous attempts to develop molecular imaging approaches, there is still no widely-accepted and reliable method for *in vivo* imaging of apoptosis. We hypothesized that the distinct cellular morphologic changes associated with treatment-induced apoptosis, such as cell shrinkage, cytoplasm condensation, and DNA fragmentation, can be detected by temporal diffusion spectroscopy imaging (TDSI). Cetuximab-induced apoptosis was assessed *in vitro* and *in vivo* with cetuximab-sensitive (DiFi) and insensitive (HCT-116) human colorectal cancer cell lines by TDSI. TDSI findings were complemented by flow cytometry and immunohistochemistry. Cell cycle analysis and flow cytometry detected apoptotic cell shrinkage in cetuximab-treated DiFi cells, and significant apoptosis was confirmed by histology. TDSI-derived parameters quantified key morphological changes including cell size decreases during apoptosis in responsive tumors that occurred earlier than gross tumor volume regression. TDSI provides a unique measurement of apoptosis by identifying cellular characteristics, particularly cell shrinkage. The method will assist in understanding the underlying biology of solid tumors and predict tumor response to therapies. TDSI is free of any exogenous agent or radiation, and hence is very suitable to be incorporated into clinical applications.

## Introduction

Apoptosis, or a programmed cell death, is the primary mechanism by which unwanted cells are selectively eliminated during growth, differentiation, and maintenance of tissue homeostasis. Excessive apoptosis can result in progressive loss in tissue functionality. Insufficient apoptosis allows immunologically competent cells to survive and to damage healthy organs. Significant loss of normal apoptosis can lead to excessive cell proliferation and subsequent tumor development^1,2^. One of the major mechanisms involved in anti-cancer treatments is the iatrogenic induction of apoptosis by radiation, chemotherapy, or both. Increased numbers of apoptotic cells are observed in tumors that are responsive to therapies, and an early apoptotic response has been strongly correlated with subsequent disease outcome^3–5^. Given the central role of apoptosis in cancer biology, it is desirable to have a non-invasive imaging method to monitor this process in cancer patients receiving conventional radio and/or chemo therapies as well as for the development of novel therapies.

Molecular imaging has been considered a promising approach for the detection of treatment-induced apoptosis *in vivo*. Phosphatidylserine (PS) externalisation is one of the hallmarks of cells entering apoptosis so PS-targeted radioligand imaging using agents such as Annexin-V has been proposed for *in vivo* studies of apoptosis^6–9^. However, cells dying by necrosis also bind with PS-targeted radioligands due to cell membrane breakdown, which makes *in vivo* discrimination between apoptosis and necrosis using PS-targeted radioligand challenging. Alternative tracers for imaging apoptosis target the caspase cascade^10^, but caspases also have non-apoptotic roles in several biological events, such as macrophage activation and differentiation^11,12^. Optical imaging has been shown to provide real-time imaging of therapy-induced apoptosis^13,14^, but suffers poor penetration into biological tissues, limiting its applicability to superficial tumors. High-frequency ultrasound imaging may characterize apoptosis in real-time by detecting sub-cellular nuclear changes^15,16^ but it cannot be used in many tumor sites due to its low focal detection range in tissues. Magnetic resonance spectroscopy (MRS) can detect metabolic changes that occur during the apoptotic process^17–19^, but it is limited by low temporal and spatial resolution. Magnetic resonance imaging (MRI) is one of the most widely used clinical imaging modalities with excellent soft-tissue contrast and high spatial resolution^20–22^. However, MRI has poor sensitivity for detecting specific molecular species, relying instead on detecting factors that affect the gross properties of tissue water. Thus, currently available imaging modalities suffer different, but significant, limitations, and consequently there is no reliable, clinically-available imaging method for assessing treatment-induced apoptosis *in vivo*.

Apoptosis is a highly regulated and controlled process with characteristic cellular morphologic changes, such as cell membrane blebbing, cell shrinkage, nuclear fragmentation, chromatin condensation, and DNA fragmentation. These morphologic changes are distinct from those that arise in necrosis, and hence have been considered as hallmarks of apoptosis^23^. Diffusion-weighted MRI (DWI) provides a unique means to probe tissue microstructure and hence may be sensitive to apoptosis-associated changes. However, conventional DWI quantifies diffusion in terms of an apparent diffusion coefficient (ADC), which has been found widely useful for monitoring anti-cancer therapies^24,25^ but lacks specificity as it is influenced by several factors including the tortuosity of extracellular spaces, tissue cellularity, and the integrity of cellular membranes^22^. Thus DWI cannot detect apoptosis specifically.

Temporal diffusion spectral imaging (TDSI) as described in our earlier reports^26–31^ provides an alternative approach to using DWI to assess tissue microstructure more comprehensively, and may have a better sensitivity and specificity than conventional DWI for assessing treatment-induced apoptosis. TDSI measures ADC values over a wide range of effective diffusion times, corresponding to diffusion distances ranging from sub-cellular to cellular levels (~3–20 µm). Temporal diffusion spectra plot ADC values versus the reciprocal of diffusion time (or frequency). Typical spectra for solid tumors, consisting of tightly packed cells, are characterized by a low ADC value near zero frequency (long diffusion time), followed by a rapid increase that curves to a plateau value at very high frequency (short diffusion time). Water in cellular tissues is unable to move freely because of restrictions imposed by cell membranes and intracellular organelles, spanning a range of spatial scales. Changes in the average spacing of restrictions to free diffusion largely affect the initial rise in ADC, whereas the asymptotic value reflects the average intrinsic rate of diffusion within cellular compartments prior to encountering membranes. A simplified model can be used to describe this behavior, incorporating parameters associated with cell size, extracellular-space tortuosity, and intracellular diffusion rates. These quantities can be extracted by fitting measured ADC spectra to the model and are proposed as a means to quantitatively describe typical morphological changes associating with apoptosis. For example, Supplemental Table 1 summarizes the expected changes in model parameters corresponding to cell morphological changes during apoptosis.

In the current study, we hypothesized that the distinct cellular morphologic changes such as cell shrinkage, cytoplasm condensation, and DNA fragmentation can be characterized using TDSI. This hypothesis was tested and validated using well-characterized cell cultures and *in vivo* experiments.

## Materials and Methods

### Cancer model and treatment

Cetuximab is an epidermal growth factor receptor (EGFR) inhibitor used for treatment of colon cancers with wild-type KRAS gene. Two types of human colorectal cell lines, DiFi, provided by Dr. Robert J. Coffey (Vanderbilt University Medical Center), and HCT116, obtained from American Type Culture Collection (ATCC number CCL-227), were used to create tumors in mice. It has been reported that cetuximab induces significant apoptosis in DiFi tumors, while HCT116 is not responsive to cetuximab^32,33^.

### Cell preparation

DiFi and HCT116 cell lines were cultured in RPMI Medium 1640 supplemented with 10% FBS, 50 Units/ml penicillin, and 50 g/ml streptomycin (Invitrogen, CA) under standard culture conditions in a humidified incubator maintained at 5% CO_2_ and 37 °C. DiFi and HCT116 cells were cultured in 150 mm dishes to full confluence, and then treated with cetuximab (20 nM) for 0, 24, and 48 hours. All the cells were harvested by trypsinization, washed with PBS buffer and resuspended with PBS/0.1% BSA. In MR experiments, cells were fixed with 4% paraformaldehyde in PBS for over 2 hours. After fixation, cells were washed, and approximately 35 × 10^6^ cells were centrifuged at 2000 g × 2 minutes in a 0.65 ml of Eppendorf tube to obtain a tight cell pellet. All the liquid on the top was carefully removed, and the tube with cell pellet was used for MRI measurements. All MRI acquisitions and subsequent data analyses were performed blinded to the nature of the cells or their treatments.

For flow cytometry experiments, cells were fixed with 3x volume of cold (−20 °C) absolute ethanol and kept at −20 °C. Before flow cytometry analysis (FACS), the cells were spun down and washed twice with PBS, and resuspended with propidium iodide (PI) staining solution (3.8 mM sodium citrate, 50 μg/ml PI, 0.5 mg/ml RNase A in PBS) to a final cell concentration about 1 × 10^6^ cell/ml, and incubated 3 or more hours at 4 °C before FACS analysis. Samples were analyzed by the Vanderbilt Flow Cytometry Core on a flow cytometer FACSCalibur (BD Biosciences, Mountain View, CA) with a minimum of 30,000 cells analyzed per sample. The data were collected with CellQuest software (BD Biosciences) and analyzed with Matlab R2013b.

### *In vitro* MRI measurements

All measurements were performed on a Varian DirectDrive^TM^ horizontal 4.7 T magnet (Varian Inc. Palo Alto, CA) equipped with a self-shielded SGRAD 115/60/S gradient system (Magnex Scientific Limited, Yarnton, Oxford, UK). A 40 mm inner diameter millipede volume coil was used for radiofrequency (RF) transmission and reception. As described above, DiFi and HCT116 cells were treated with cetuximab for 0, 24, and 48 hours. ADC values for both conventional DWI (using a pulsed gradient spin echo (PGSE) sequence) as well as for TDSI (using oscillating gradient spin echo (OGSE) acquisitions) were measured. Two gradient b-values (0, and 400 sec/mm^2^) were used in both PGSE and OGSE measurements. The OGSE pulse sequence used two apodized cosine-modulated gradients as described in^34^. For PGSE experiments, diffusion gradient durations were δ = 4 ms, and separation Δ = 48 ms. The OGSE measurements were made using gradient frequencies from 50 to 350 Hz with an interval of 50 Hz. Note that the echo times (TE = 67 ms) for both PGSE and OGSE measurements were the same to minimize different relaxation effects.

### Animals and tumor induction

Female Athymic nude mice (Harlan Laboratories, Inc., Indianapolis, IN) were used for the study and observed daily and weighed weekly to ensure that interventions were well tolerated.

### Ethics statement

The use of mice was conducted in accordance with the regulations set forward by the national animal protection committees and in accordance with the U.S. Association for Laboratory Animal Care recommendations for the care and use of laboratory animals. All the techniques/procedures have been refined to provide for maximum comfort/minimal stress to the animals. Experiments performed at Vanderbilt University Institute of Imaging Science have been approved by the Vanderbilt University Institutional Animal Care and Usage Committee (protocol number M1800025).

### *In vivo* experiment outline

DiFi and HCT116 xenografts were generated in female Athymic nude mice (Harlan Sprague-Dawley) following subcutaneously injection of 1 × 10^6^ to 2 × 10^6^ cells. Day 0 is defined for each mouse as the time point when the tumor reached a size of 200–300 mm^3^. 3 DiFi-bearing and 3 HCT116-bearing mice were sacrificed at Day 0 for baseline histology. 10 DiFi-bearing mice received 2 treatments of either cetuximab (n = 5, 10 mg/kg) or PBS (n = 5, 10 mg/kg) at Day 0 and 2. Another 10 DiFi-bearing mice received 4 treatments of either cetuximab (n = 5, 10 mg/kg) or PBS (n = 5, 10 mg/kg) at Day 0, 2, 4, and 6. HCT116-bearing mice were divided into 2 cohorts (n = 4). Cohort #1 received 2 treatments of cetuximab (10 mg/kg) at Day 0 and 2. Cohort #2 received 4 treatments of cetuximab (10 mg/kg) at Day 0, 2, 4, and 6. The injection of drug or drug vehicle was administered by intraperitoneal injection. Each mouse treated with either cetuximab or PBS was imaged pre-treatment at Day 0 and post-treatment at Days 4 and 8, and was sacrificed for collection of tumor tissues immediately after final imaging. Detailed MR imaging time schedule for drug vehicle and cetuximab treatment groups is shown in Supplemental Table 2.

All MRI acquisitions and subsequent data analyses were performed blinded to the nature of the mice or their treatments.

### *In vivo* MR imaging

Tumor-bearing mice were anesthetized with a 2%/98% isoflurane/oxygen mixture before and during MR scanning and the magnet bore temperature was kept at 32 °C using a warm-air feedback system. Stretchable tapes were used to maintain the proper positioning of hind limbs and tumors and to reduce motion-induced artifacts in the MR data. Respiratory signals were monitored using a small pneumatic pillow placed on top of the mouse back and respiration gating (SA Instruments, Stony Brook, NY) was applied to further reduce motion artifacts. A thin tube filled with an aqueous solution of 5 mM CuSO_4_ was placed beneath the mouse in thermal equilibrium with the magnet bore as a reference sample to ensure its ADC value remained constant for all measurements.

Both OGSE and PGSE sequences were implemented with a respiratory-gated, fat-saturated, 2-shot echo planar imaging (EPI) acquisition, using the same hardware as used in the *in vitro* MRI measurements described above. The imaging parameters for PGSE acquisitions are diffusion gradient duration δ = 4 ms, and separation Δ = 48 ms. The OGSE sequences used gradient frequencies from 50 to 300 Hz with δ/Δ = 20/25 ms, corresponding to effective diffusion times roughly from 5 to 0.8 ms. Two b values (0, and 400 sec/mm^2^) were used for all diffusion acquisitions. Multiple axial slices covering the entire tumor were acquired with a slice thickness of 2 mm. The data matrix size was 128 × 64 with FOV = 40 × 20 mm, yielding an isotropic in-plane resolution of 312.5 um. Note that the echo times (echo time = 67 ms) for all PGSE and OGSE measurements were the same to reduce relaxation effects.

### Immunohistochemistry

All the mice were sacrificed immediately following the last imaging session. The tumors were then harvested, cut into small pieces of approximately 2 mm in thickness and immersed in 10% neutral buffered formalin for 24 hours. Tumor tissues were embedded in paraffin and sectioned (8 µm thickness) and stained with either cleaved-caspase-3 antibody to measure apoptotic cells or Na^+^/K^+^-ATPase (ab76020, Abcam) to delineate cell membranes. Briefly, tissue samples were de-paraffinized, rehydrated, and antigen retrieval was performed using pH 6.1 citrate buffer (S169984-2, Dako) for 20 minutes at 105 °C in a pressure cooker followed by a 10 minute bench cool down. Samples were treated with 3% hydrogen peroxide, and blocked for 30 minutes in PBS/3% bovine serum albumin/10% donkey serum. Primary Na^+^/K^+^-ATPase antibody was incubated overnight at 4 °C followed by Cy7 conjugated anti-rabbit secondary antibody and DAPI. Caspase-3 and Na^+^/K^+^-ATPase stained slides were scanned by a Leica SCN400 Slide Scanner and an Aperio Versa 200 Slide Scanner, respectively, at 20x to generate high-resolution digital images.

### Diffusion models and data analysis

Tumor ROIs were identified as hyper-intense regions on T2 weighted MR images, and for each voxel within each ROI ADC values at different frequencies (effective diffusion times) were calculated using Eq. (1):

1 $${\rm{ADC}}=-\,\mathrm{log}(\frac{I({b}_{2}=400)}{I({b}_{1}=0)})/({b}_{2}-{b}_{1})$$

Here I(b) is the diffusion-weighted signal intensity with a specific b value (here, 0 or 400 sec/mm^2^).

Actively growing tumors can be modeled as tightly packed, impermeable, spherical cells. We used an analytical expression, reported in^29^, that relates ADC to sphere diameter for measurements using different oscillation frequencies *f*.

ADC values in impermeable spheres are given by:

2 $$\begin{array}{rcl}AD{C}_{sphere}({\rm{f}}) & = & \frac{8{\pi }^{2}{f}^{2}}{\delta }\sum _{n}\frac{{B}_{n}{\lambda }_{n}^{2}({D}_{f\to inf}-{D}_{f\to 0})}{{({\lambda }_{n}^{2}{({D}_{f\to inf}-{D}_{f\to 0})}^{2}+4{\pi }^{2}{f}^{2})}^{2}}\\  &  & \frac{({\lambda }_{n}^{2}{({D}_{f\to inf}-{D}_{f\to 0})}^{2}+4{\pi }^{2}{f}^{2})}{{\lambda }_{n}{D}_{in}}[\frac{\delta }{2}+\frac{\sin (4\pi f\delta )}{8\pi f}]-1\\  &  & +\,\exp (-{\lambda }_{n}\delta ({D}_{f\to inf}-{D}_{f\to 0}))\\  &  & +\,\exp (-{\lambda }_{n}{\rm{\Delta }}({D}_{f\to inf}-{D}_{f\to 0}))(1-\,\cosh ({\lambda }_{n}\delta ({D}_{f\to inf}-{D}_{f\to 0})))\end{array}$$

f is the oscillation frequency, δ is the gradient duration, Δ is the separation of two diffusion gradients. The terms B_n_ and λ_n_ are structure dependent parameters that depend on sphere diameters^29^. This theoretical expression has been validated by computer simulations^29^ and experiments on model systems^35^. The predicted ADC drops to zero at very long diffusion times or low frequencies due to complete restriction of water motions. However, in biological tissues the presence of permeable cell membranes and the nature of the extracellular space tortuosity lead to a non-zero value. To account for this, a modified ADC model was used as

3 $$AD{C}_{tumor}(f)=AD{C}_{sphere}(f,d,{D}_{f\to inf}-{D}_{f\to 0})+{D}_{f\to 0}$$

where D_f→inf_ and D_f→0_ represent the tumor ADC values at infinitely high and zero frequencies, respectively. The mean ADC spectra of the tumor region on each slice were fit to this equation. The restriction parameter *d* represents the mean dimension between diffusion barriers within the tumor, which is a reflection mainly of the mean tumor cell size as cell membranes have been reported to be the dominant restriction barriers in tissues^36^. In tumors, D_f→0_ and D_f→inf_ are mainly determined by the extracellular tortuosity^37^, and intracellular diffusion coefficient respectively.

For tumor tissues dominated by very late stage apoptotic cells, water diffusion is no longer restricted due to a loss of membrane integrity and even loss of cells. ADC values measured at different effective diffusion times are then expected to be constant.

For voxels to be fit using our TDSI model, the three unknown variables (d, D_f→inf_, and D_f→0_) were estimated using the lsqcurvefit function in Matlab (Mathworks, Natick, MA). The constraints for fitting parameters were based on physiologically relevant values: 0 ≤ d ≤ 40 μm, 0 ≤ D_f→inf_ ≤ 3.0 µm^2^/ms, 0 ≤ D_f→0_ ≤ 3.0 µm^2^/ms. Randomly-generated initial parameter values were used in the fittings.

### Statistical analysis

**Figure 1:**
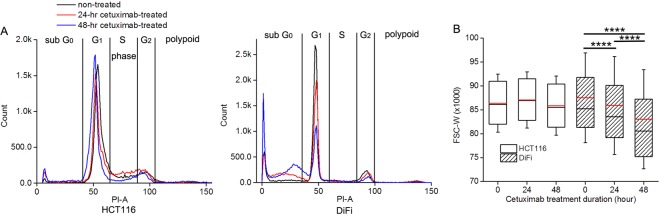
Flow cytometry analyses indicate the occurrence of apoptosis in cetuximab-treated DiFi cell pellets and a corresponding decrease in cell size. (**A**) Histograms of the emission light intensity of PI labelled cells (PI-A). (**B**) Box-and-whisker plots of the FSC-W for DiFi and HCT116 cell pellets treated with 20 nM cetuximab for 0, 24, and 48 hrs. The sample size of each cohort is 10000. For all the Box-and-whisker plots, the 25^th^–75^th^ percentiles are blocked by the box, the black and red bands inside the box are the median and mean, respectively, and the whiskers mark the SD. ****P < 0.0001 as measured by one-way ANOVA with a FDR (False Discovery Rate) posttest.

**Figure 2:**
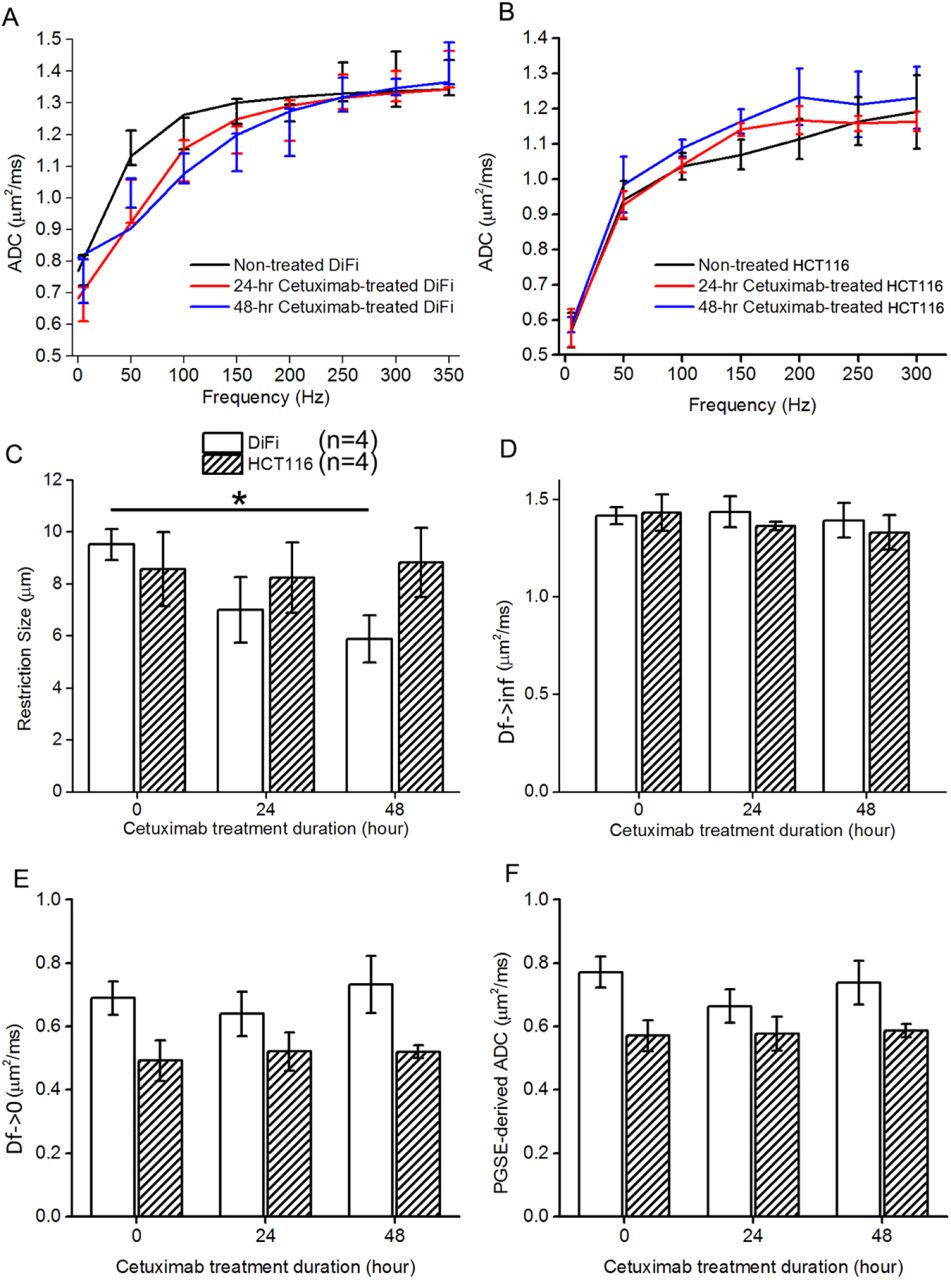
TDSI-derived restriction size detects decreases in cell size for cetuximab-treated DiFi cells. (**A,B**) Fitted d (**A**), D_f→inf_ (**B**), D_f→0_ (**C**), and PGSE-derived ADC (**D**) for DiFi and HCT116 cell pellets treated with 20 nM cetuximab for 0, 24, and 48 hours. *P < 0.05 as measured by one-way ANOVA with a FDR (False Discovery Rate) post test.

An F-test^38^ was conducted to determine which model (constant ADCs vs. tumor ADC model described in Eq. (2)) is statistically better at describing the ADC spectra on a voxel wise basis. Differences in cell sizes derived from FACS, and MR-derived measures of restriction size between the two types of cell samples with or without Cetuximab treatment (Figs 1, 2) were summarized using means and standard deviations, and compared by two-way analysis of variance (ANOVA). The percentage changes in three fitted parameters from the TDSI data (d, D_f→inf_, and D_f→0_), tumor volume and conventional (PGSE-derived) ADC values for the two types of tumors treated with either PBS or Cetuximab (Fig. 4) were also summarized using means and standard deviations, and compared by two-way analysis of variance (ANOVA). All the tests were two-sided and an FDR (False Discovery Rate) adjusted p-value of 0.05 or less was taken to indicate statistical significance. Statistical analyses were performed using Matlab R2013b.

## Results

### *In vitro* experiments

#### Cell cycle analysis and flow cytometry detects apoptotic cell shrinkage in cetuximab-treated DiFi cells

The emission light intensity of propidium iodide labelled cells (PI-A) is proportional to the amount of DNA present in the cells, so the percentage of cells in different phases of the cell cycle can be calculated from the histogram of PI-A^39^. As shown Fig. 1A, the DNA content of G_2_/M cells is about twice that of G_0_/G_1_ cells because the chromosomes have doubled during S phase. Polyploidy frequently appears in cancer cells and exhibits greater DNA content than G_2_/M cells. DNA fragmentation is one of the hallmarks of apoptosis^40^. As a result, sub G_0_ cells mainly consist of apoptotic cells. It is clear that the percentage of apoptotic cells (sub G_0_ cells) in DiFi cells increases dramatically with prolonged duration of cetuximab treatment (Supplemental Table 3). Over half the DiFi cells receiving 48-hour treatment underwent apoptosis, indicating that DiFi are highly sensitive to cetuximab treatment. On the other hand, the proportion of apoptotic cells in HCT116 cells increased only slightly following cetuximab treatment, indicating drug resistance.

The width of forward-scattered light (FSC-W) is proportional to cell size^39^. As shown in Fig. 1B, FSC-Ws for DiFi cells decrease continuously as the treatment is given for longer (p < 0.0001), indicating decreases in cell size during apoptosis. No significant differences (p > 0.05) in FSC-W are observed among cetuximab-treated HCT116 cells with different treatment durations.

#### TDSI-derived restriction size quantifies apoptotic cell shrinkage

The mean ADC spectra (n = 4) for DiFi and HCT116 cell pellets treated with 20 nM cetuximab for 0, 24, and 48 hours are shown in Fig. 2A,B. A typical ADC spectrum for tumor is characterized by a low ADC value at zero frequency followed by a rapid increase that reaches a plateau, quantified by the three spectral parameters D_f→0_, d and D_f→inf_ respectively. In particular, changes in restriction size lead to variations in the shape of the ADC spectrum. In Fig. 2A, the mean ADC spectrum for non-treated DiFi pellets becomes almost flat when the frequency is larger than 150 Hz, while spectra for treated DiFi cell pellets reach the plateau only when the frequency is larger than 250 Hz. This slower increase in ADC values with frequency is due to cell shrinkage. By contrast, the mean ADC spectra for both treated and non-treated HCT116 cell pellets are almost parallel over the initial segment of the curves, suggesting the restriction size stays the same.

The fitted spectral parameters for both treated and non-treated DiFi and HCT116 cell pellets are summarized in Fig. 2C–F. The mean restriction size of DiFi cells shows a decreasing trend with increasing duration of cetuximab treatment. The mean restriction size of DiFi cells receiving 48-hour treatment is significantly smaller than that of non-treated DiFi cells (p = 0.04). By contrast, there are no significant changes in restriction size of HCT116 cells (p > 0.05). The decrease in restriction size for cetuximab-treated DiFi cells reflects cell shrinkage during treatment-induced apoptosis, which is consistent with the cell cycle analyses (Fig. 1A & Supplemental Table 3) and changes in FSC-W measured by flow cytometry (Fig. 1B).

There were also changes in D_f→0_ and PGSE-derived ADC between cells receiving different dosages of treatment, indicating treatment-induced changes in tortuosity of the extracellular space and possibly overall cell density. In our experiments, all the cells were centrifuged to create tight pellets using the same speed and time. Morphological properties of the extracellular space depend on how the cells pack during centrifugation, and may correlate with the cell size distribution. Therefore, it is difficult to correlate changes in D_f→0_ and PGSE-derived ADC directly with changes in mean cell sizes. Cell pellets are not appropriate for assessing treatment-induced changes in tortuosity of the extracellular space, but they fulfill our purpose of measuring treatment-induced changes in cell size.

### *In vivo* experiments

#### TDSI-derived parameters quantify key morphological changes during apoptosis in responsive tumors that occur earlier than gross tumor volume regression

**Figure 3:**
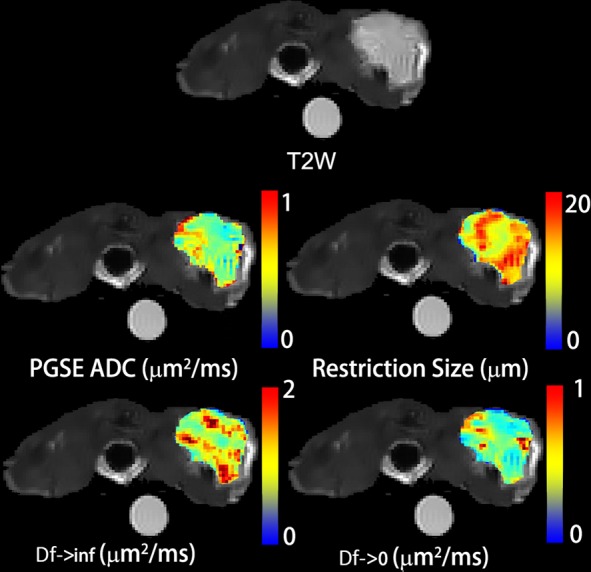
PGSE-derived ADC map, and TDSI-derived parametric maps (restriction size d, D_f→inf_, and D_f→0_) of a representative slice through a non-treated DiFi tumor, overlaid on T2-weighted MR images.

A PGSE-derived ADC map, and TDSI-derived parametric maps (of restriction size d, D_f→inf_, and D_f→0_) of a representative slice through a non-treated DiFi tumor are shown in Fig. 3. All the maps exhibit significant heterogeneity. The PGSE-derived ADC shows similar patterns to the TDSI-derived D_f→0_ map, due to their similar biophysical interpretations. The restriction map is different from other maps, indicating that it provides unique microstructural information. A detailed comparison, histograms of the PGSE-derived ADC and three TDSI-derived parameters for the same tumor (either cetuximab-treated DiFi, PBS-treated DiFi, or cetuximab-treated HCT116) at day-0/baseline (no treatment), day-4 (2 treatments), and day-8 (4 treatments) are displayed in Supplemental Fig. 1. For the cetuximab-treated DiFi tumor, there is an evident shift in the restriction size peak towards lower restriction sizes at day-4 and day-8, while the distribution of PGSE-derived ADC starts to shift to higher ADC at day-8.

**Figure 4:**
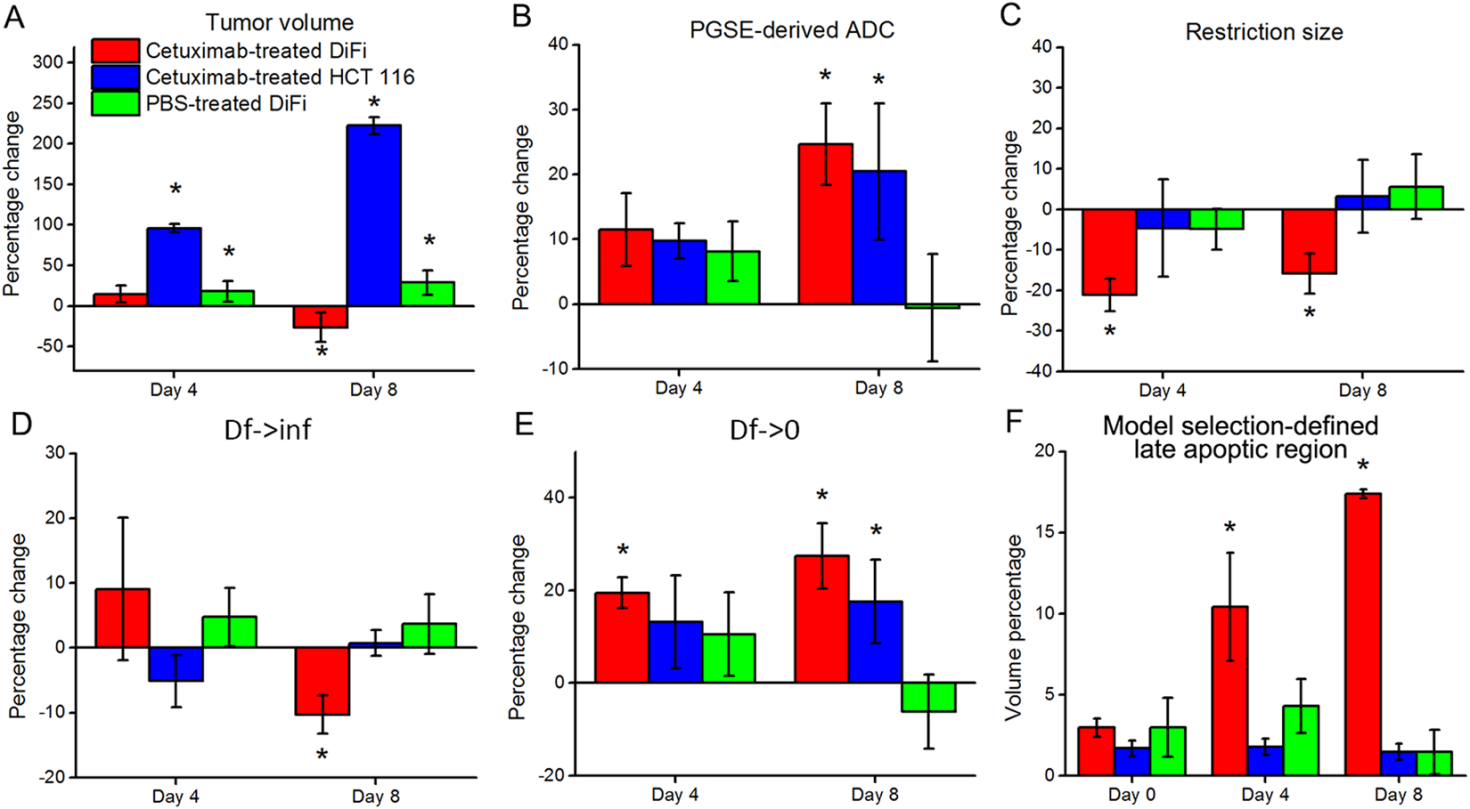
(**A**–**E**) Percentage changes in tumor volume (**A**), ADC obtained from PGSE (**B**), and three fitted spectral parameters (*d*, D_f→inf_, and D_f→0_) for cetuximab/PBS-treated DiFi and cetuximab-treated HCT116. (**F**) Volume fractions of late-stage apoptotic regions defined by model selection between the constant ADC model and tumor ADC model described in Eq. 3. *P < 0.05 as measured by one-way ANOVA with a FDR (False Discovery Rate) posttest.

Figure 4A–E summarizes percentage changes in tumor volume, three fitted spectral parameters (*d*, *D*_*f→*inf_, and D_f→0_), and ADC values obtained with PGSE acquisitions for DiFi and HCT116 tumors at day-4 (2 treatments) and day-8 (4 treatments). Tumor volume for the cetuximab-treated, DiFi-bearing mice decreased significantly at the late (Day 8) time point, indicating the efficacy of cetuximab in the treatment of DiFi tumors. As expected, the tumor growth for the other two cohorts is not mitigated by the treatment at both Days 4 and 8.

For the cetuximab-treated DiFi tumors, the mean restriction size, *d*, decreased significantly at Day 4 (p = 0.001) and 8 (p = 0.002), corresponding to treatment-induced cellular shrinkage. *D*_*f→*inf_ decreased at Day 8 (p = 0.001) indicating slower diffusion inside the cetuximab-treated DiFi cells, probably corresponding to the effects of nuclear fragmentation and cytosolic condensation. D_f→0_ for both cetuximab-treated DiFi- and HCT116-bearing mice was significantly increased (p < 0.05) at both Days 4 and 8, however, resulting from different biological processes. The former is associated with a decrease of extracellular tortuosity which may result from treatment-induced cell death, while the latter indicates an increase of fluid content likely associated with the unconstrained development of the HCT116 tumors. The changes in ADC values measured by the PGSE sequence with a long diffusion time are very similar to the changes in D_f→0_ except that the PGSE-derived ADC values for cetuximab-treated DiFi tumors show an increasing trend without reaching statistical significance. The three fitted spectral parameters for cetuximab-treated DiFi tumor tissues all changed significantly at Day 4, which is earlier than the observed change in tumor volume.

**Figure 5:**
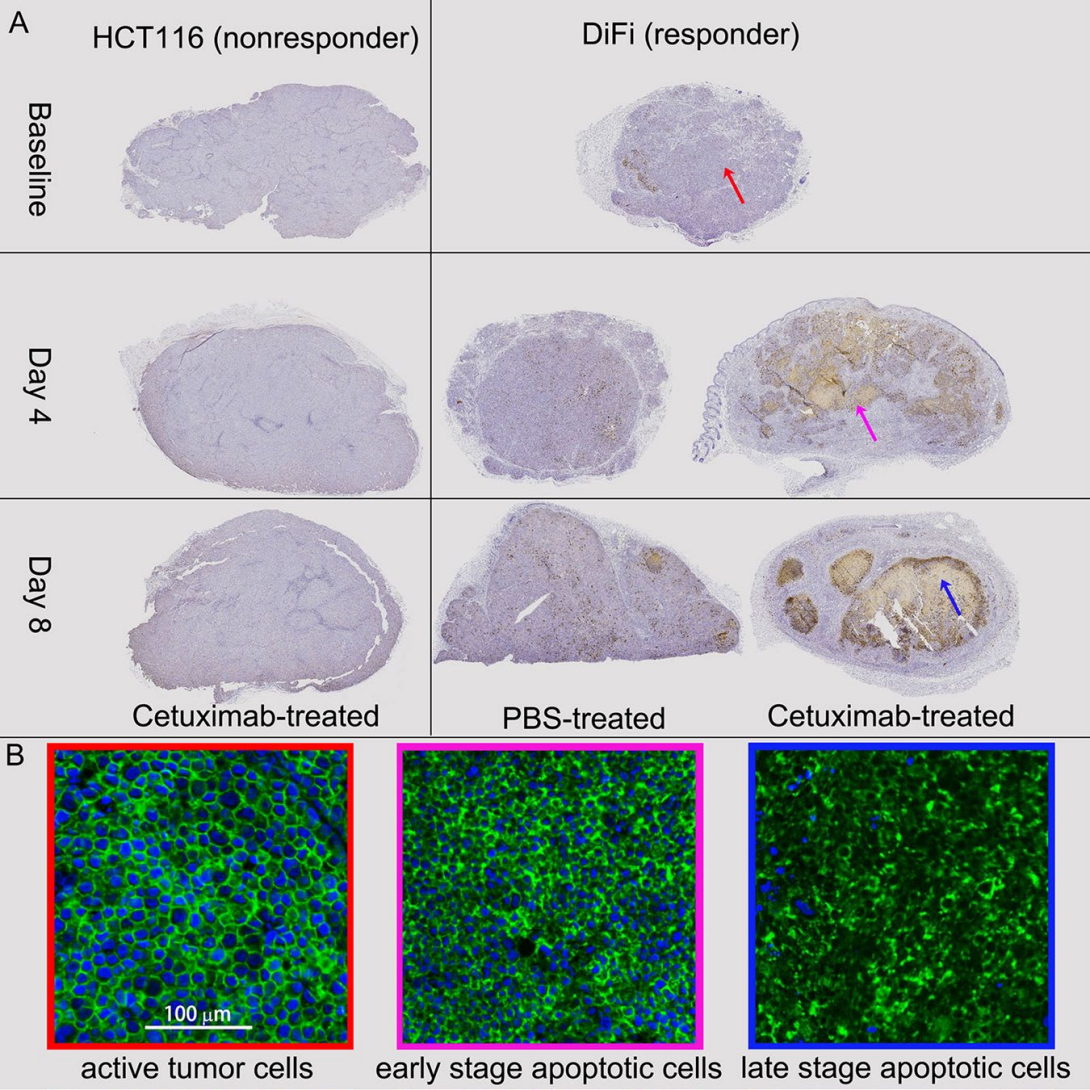
(**A**) Caspase-3 stained histological images of DiFi tumor tissues treated with PBS and Cetuximab at day-0 (no treatment), day-4 (2 treatments), and day-8 (4 treatments), x2. (**B**) Na+/K+-ATPase stained histological images for tissue regions consisting either of active tumor cells, early stage apoptotic cells, or late stage apoptotic cells. Nuclei were visualized with DAPI (blue). Note that all the cells express high level, relative evenly distributed Na+/K+-ATPase (green) on their plasma membranes.

As discussed previously, the extraction of parameters from the ADC spectra relies on the degree of curvature of the spectra. If the voxel data are dominated by signals from late-stage apoptotic cells, the ADC spectra are almost flat because then the loss of cell membrane integrity reduces the effects of restriction. A voxel-based selection between the constant ADC model and tumor ADC model (Eq. 2) is thus hypothesized to differentiate regions of late-stage apoptotic cells from regions of still viable tumor cells. As shown in Fig. 5F, the volume fractions of the late-stage apoptotic regions in cetuximab-treated DiFi tumors increased dramatically at day-4 and day-8, suggesting that it may be an early indicator of treatment responsiveness.

#### Histology shows that cetuximab induces significant apoptosis in DiFi tumors

Figure 5A compares typical cleaved caspase-3 stained DiFi/HCT116 tumor slices treated with PBS/cetuximab at baseline (no treatment), day 4 (2 treatments), and day 8 (4 treatments). As suggested by the images, there are no apparent differences in cleaved caspase 3 immunoreactivity between the three time points for PBS-treated DiFi tumor and cetuximab-treated HCT116 tumor tissues. For cetuximab-treated DiFi tumor tissues, increased cleaved-caspase-3 staining is observed extensively at day 4 and day 8. A summary of apoptotic index (AI) values for DiFi and HCT116 tumors treated with either cetuximab or PBS are shown in Supplemental Table 4. The AI was calculated as a percentage of the identifiable cells exhibiting staining out of the total number of cells examined on the sections stained with cleaved-caspase-3. Apoptotic cells that were present in areas where intense necrosis had occurred were excluded from the analysis. The AIs for 4-day and 8-day cetuximab-treated DiFi tumors are 31.9 ± 7.3% and 28.8 ± 6.5%, respectively, significantly (p < 0.001) higher than that for baseline DiFi tumors (7.0 ± 1.5%). Figure 5B visualizes the cell membranes in green with immunofluorescence staining for Na^+^/K^+^-ATPase, providing a better depiction of the morphologies of DiFi tumor cells with and without treatment. The actively growing tumor cells (red panel) are large, round, and tightly packed, while the early-stage apoptotic cells (pink panel) have much smaller nuclei and cell sizes. The late-stage apoptotic regions (blue panel) mainly consist of membrane-bound apoptotic bodies.

## Discussion

Apoptosis has been commonly exploited as a therapeutic target in a variety of chemotherapy and radiotherapy regimens. *In vivo* monitoring of treatment-induced apoptosis is of practical significance in monitoring treatments in both preclinical and clinical applications. Different from conventional molecular imaging that requires exogenous agents that are typically radioactive, diffusion MRI is free of exogenous agents and radiation, which is ideal for translation to clinical practice. Although conventional measures of ADC at single diffusion times have been shown to be sensitive to some morphological changes associated with apoptosis, they are not specific for apoptosis because single ADC values are affected by several other factors, especially tissue cellularity and the presence of necrosis. It has been suggested that a combination of multiple ADC values measured with different diffusion times provides a more precise characterization of tumor microstructure^41–43^ but the selection of diffusion times needs to be appropriate for the spatial scales over which relevant structural changes occur. For cellular scales the diffusion times need to be substantially shorter than typically available using conventional DWI. In this study, we developed TDSI using short diffusion times as an extension of diffusion MRI, and showed that (i) the appearance of ADC spectra can differentiate late apoptotic regions that consist of damaged cells, cell debris, and necrotic regions from actively growing tumor regions that consist of tightly packed tumor cells with intact membranes; and (ii) three parameters (d, D_f→inf_, and D_f→0_) extracted from ADC spectra have a strong correlation with typical morphological changes characteristic of apoptosis. For example, the restriction size *d* for cetuximab-treated DiFi tumors decreased significantly after the first two treatments and decreased even more after further treatments, indicating apoptotic cell shrinkage. Nuclear condensation and fragmentation slow the water diffusion within the cells^44^, and therefore lowers D_f→inf_ for cetuximab-treated DiFi tumors receiving four treatments. D_f→0_ is sensitive to cell density changes as a result of cell death. The combination of these spectral parameters (d, D_f→inf_, and D_f→0_) may provide a specific *in vivo* assessment of treatment-induced apoptosis in solid tumors. In addition, changes reported by these spectral parameters occurred earlier than changes in conventional tumor size measurements, suggesting that TDSI could be used as an early *in vivo* biomarker for tumor responsiveness following therapies targeting apoptosis.

Treatment induced apoptosis is usually accompanied by necrosis, another major form of cell death. TDSI is hypothesized to differentiate early apoptosis from necrosis/late stage apoptosis due to their different morphologies. During the early process of apoptosis, cells shrink with condensed cytoplasm, which is expected to decrease TDSI-derived restriction size (d) and intracellular diffusivity (D_f→inf_). The cell membrane permeability has been reported to increase during apoptosis, leading to an increase of water mobility and increased ADC. However, our previous study has shown that the measurements of restriction size are not affected by the variations of cell membrane permeability^45^. During the later stages of apoptosis, cells are separated into several apoptotic bodies. Cellular death due to necrosis results in the loss of cell membrane integrity and an uncontrolled release of cellular contents into the extracellular space^46^. Such changes might be expected to decrease the length scales of the restrictions that hinder/restrict tissue water movement, which may also lead to a decreasing averaging restriction size measured by TDSI. However, as TDSI emphasizes the effects of restrictions in a specific size range (e.g., 3–20 μm in this study), necrotic bodies and fragments of cellular debris are too small to influence the restriction size measurements. Thus water diffusion in necrotic/late apoptotic regions appears to be relatively ‘unrestricted’ and ADC values do not show any diffusion time dependency within the diffusion time range we used in the current study. We used a voxel-based statistical model-selection (tumor ADC model vs. constant ADCs) to differentiate imaging voxels dominated by necrosis/late apoptosis from those dominated by actively growing cells and/or early stage apoptotic cells. For imaging voxels containing both apoptosis and necrosis, necrosis/late apoptosis does not contribute to the diffusion time dependency of ADC and thus, has minimal bias on early apoptosis quantification. The primary goal of this study is to test the ability of TDSI for assessing early cellular changes associated with treatment-induced apoptosis. For the next step, a more complex two-compartmental model (e.g., intra- and extracellular) and diffusion measurements with more diffusion times and b-values can be included to obtain the intact cell volume fraction, which will potentially allow more insights into the fractions of cells undergoing apoptosis/necrosis.

The fitted absolute restriction sizes in non-treated tumor tissues (~8 µm) are smaller than the reported cell sizes for DiFi (~14 µm) and HCT-116 (~13 µm) cells. This is partly due to the confounding influence of extra-cellular diffusion, and assumptions made about the shapes of cells. The average restriction size in extracellular space is usually smaller than the average cancer cell size due to the relatively high packing density of cancer cells in tumors. This biases the estimation of TDSI-derived restriction size and make it smaller than the mean cancer cell size. Note that it is possible to apply a more complex two-compartment (intra- and extracellular) diffusion model for fitting diffusion MRI data to extract mean cancer cell size as well as intracellular volume fraction. Although this type of model has proven successful in well-characterized cell cultures^27^, fixed tissues^31^, and xenograft tumors^28^, the total scan time increases remarkably due to the requirement of multiple diffusion times and b values, which is not desirable for practical uses. However, the fitted restriction size is capable of detecting the variations of cell sizes associated with treatment-induced apoptosis. Considering the fast acquisition of TDSI (a minimum of three ADC values required), the restriction sizes obtained using TDSI provide a good indicator of mean cancer cell size. Therefore, TDSI is suitable for fast estimation of mean cancer cell size and intracellular diffusivity, although more complex multi-compartment diffusion models provide higher accuracy of estimated metrics but at a cost of longer scan time.

It is a standard protocol to fix cells with ethanol in the flow cytometry experiments. For the *in vitro* MR measurements, the fixation is necessary but does not affect our main results. Without fixation, live cells in cell pellets may undergo apoptosis or necrosis during relatively long MR measurements, which can significantly contaminate the experiments. To prevent such a contamination, we used 4% formaldehyde for 5 minutes for a snap fixation to prevent any alteration of cell status during MR experiments. The fixation itself may cause three main changes that influence diffusion measurements, i.e., cell shrinkage, cell membrane permeability change, and diffusion coefficient change due to protein crosslink. However, none of these changes affect our main results. First, although fixation cause a slight cell shrinkage^47–49^, it affects both the responsive and resistant groups in the same way. The relative size differences between groups does not change, i.e. the apoptotic cells are still smaller than normal cells even after fixation. Because we focus on the relative changes of restriction (cell) size, the fixation does not change our conclusion. Second, as shown in our previous study^45^, the change of cell membrane permeability does not affect our measurements of restriction size. Third, it is true that the crosslinking of proteins may alter local diffusion properties, but this is reflected by the change of diffusion coefficients D_f→0_ and D_f→inf_, not by restriction size. In summary, fixation provides a reliable means to stabilize cell morphology during MR experiments and its influences do not change our main results.

The microcirculatory flow effects on OGSE and PGSE diffusion measurements were assumed to be negligible in this study. We made this assumption because the perfusion fraction of tissues has been reported to be significantly smaller than the diffusion fraction of tissues (20,22). In applications where microcirculatory flow effects on ADC measurements are significant due to effects of tumor angiogenesis, ADC values can be obtained from PGSE and OGSE signals acquired at only high b values (>200 sec/mm^2^) that are not affected by microcirculatory flow. Also, we can modify the current PGSE/OGSE sequences to acquire perfusion-free signals by adding a filter with a small b value at the beginning of each sequence^50^.

There are limitations within the current study. Firstly, as discussed previously, TDSI is hypothesized to differentiate early apoptosis from necrosis/late stage apoptosis due to their different cellular morphologies. Future studies will include assessing cellular changes associated with necrosis and/or other cell death forms. This will allow more insights into the specificity of TDSI quantification of treatment-induced apoptosis. Also, larger sample sizes, more animal models, more imaging timepoints, different treatment schedules, and different dosages will be included in future studies to facilitate analysis of the repeatability and sensitivity of the proposed methodology for apoptosis assessment. Secondly, due to the challenges of co-registration between histology and MRI of deformable xenografts in mouse hind limbs, representative ROIs were selected from the histological/immunohistochemistry pictures to explain the MR observations. To provide further validation, it will be preferable to perform *ex vivo* treatment studies that allow accurate whole tumor registration between MR and histology images.

## Conclusion

This report proposes a new diffusion-based MRI method for *in vivo* imaging of treatment-induced apoptosis. Different from conventional molecular imaging, TDSI is free of exogenous agents and radiation. Although this approach should be further examined in additional tumor models, we have shown that TDSI provide a unique measurement of apoptosis by characterizing the hallmark of apoptosis (notably, cell shrinkage), instead of targeting specific molecular pathways. Although apoptosis is biologically complicated and depends on tumor type and treatment, the morphological effects, especially cell shrinkage, are common in various tumor types with different treatments. Therefore, TDSI may have a wide array of applications for *in vivo* imaging and monitoring of apoptosis in different types of tumors following chemo/radio therapies.

## Supplementary information

supplementary info
