# Supplementary material for: In vivo magnetic resonance imaging of treatment-induced apoptosis
Source: Sci Rep. 2019 Jul 2;9:9540. doi: 10.1038/s41598-019-45864-y (PMC6606573; doi:10.1038/s41598-019-45864-y)
Supplement: Supplementary file 1 — supplementary info [file 41598_2019_45864_MOESM1_ESM.pdf]

In vivo magnetic resonance imaging of treatment-induced apoptosis

Xiaoyu Jiang[1,2], Eliot T. McKinley[3], Jingping Xie[1,2], Hua Li[1,2], Junzhong Xu[1,2,4-6§], John C. Gore[1,2,4-7§]

[1] Institute of Imaging Science, Vanderbilt University, Nashville, TN 37232, USA

[2] Department of Radiology and Radiological Sciences, Vanderbilt University, Nashville, TN 37232, USA

[3] Department of Medicine, Vanderbilt University Medical Center, Nashville, TN 37232, USA

[4] Vanderbilt-Ingram Cancer Center, Vanderbilt University, Nashville, TN 37232, USA

[5] Department of Physics and Astronomy, Vanderbilt University, Nashville, TN 37232, USA

[6] Department of Biomedical Engineering, Vanderbilt University, Nashville, TN 37232, USA

[7] Department of Molecular Physiology and Biophysics, Vanderbilt University, Nashville, TN 37232, USA

[§] Equal contributions to the research; Correspondence to Junzhong.xu@vumc.org and John.gore@vumc.org

The authors declare no potential conflicts of interest.

Supplementary Material:

**Cell morphology changes during apoptosis**	**Anticipated changes in biophysical features**	**TDSI derived metrics**
Cytoplasmic condensation, DNA defragmentation	Intracellular viscosity ↑	D_{inf} ↓
Cell shrinkage	Cell size ↓	d ↓
	Extracellular tortuosity ↓	D_0 ↑
Membrane blebbing	Membrane permeability ↑	**N/A***

Supplemental Table 1. Summary of the changes in cellular morphology during apoptosis, with corresponding anticipated changes in model features and TDSI metrics. (↑: increase, ↓: decrease) *The increased membrane permeability due to blebbing has been shown to not affect the estimation of cell size and intracellular diffusivity by TDSI, but affects conventional diffusion MRI (29).

		Day 0 (baseline)	Day 4 (after 2 treatments)	Day 8 (after 4 treatments)
No. of animals for MRI	Cetuximab-treated DiFi tumors	12	10	5
	PBS-treated DiFi tumors	11	10	5
	Cetuximab-treated HCT116 tumors	11	8	4
The number of animals decreased from Day 0 to Day 8 for histological examination.				

Supplemental Table 2. MRI time schedule for drug vehicle (PBS) and cetuximab treatment groups.

		Sub G_0	G_0/G_1	S phase	G_2/M	Polypoid
DiFi	0	15.8%	66.8%	2.2%	11.7%	3.5%
	24	33.7%	52.1%	1.9%	8.9%	3.5%
	48	52.7%	38.0%	2.4%	5.2%	1.7%
HCT116	0	2.0%	72.5%	7.5%	13.2%	4.8%
	24	5.4%	62.4%	11.8%	16.0%	4.4%
	48	8.0%	73.1%	3.8%	10.5%	4.6%

Supplemental Table 3. The percentage of cells in the different phases of the cell cycle.

Apoptotic index (%)	HCT116 (nonresponder)	DiFi (responder)	
baseline	2.7±1.2	7.0±1.5	
	Cetuximab-treated	PBS-treated	Cetuximab-treated
Day 4 (2 treatments)	3.2±1.3	8.6±2.2	31.9±7.3
Day 8 (4 treatments)	2.7±1.1	10.2±3.4	28.8±6.5

Supplemental Table 4. Summary of apoptotic index (AI) for DiFi and HCT116 tumors at Day 0 (baseline), and with 2 or 4 treatments of either cetuximab or PBS. The AI was calculated as a percentage of the identifiable cells exhibiting staining out of the total number of cells examined on the sections stained with cleaved-caspase-3.

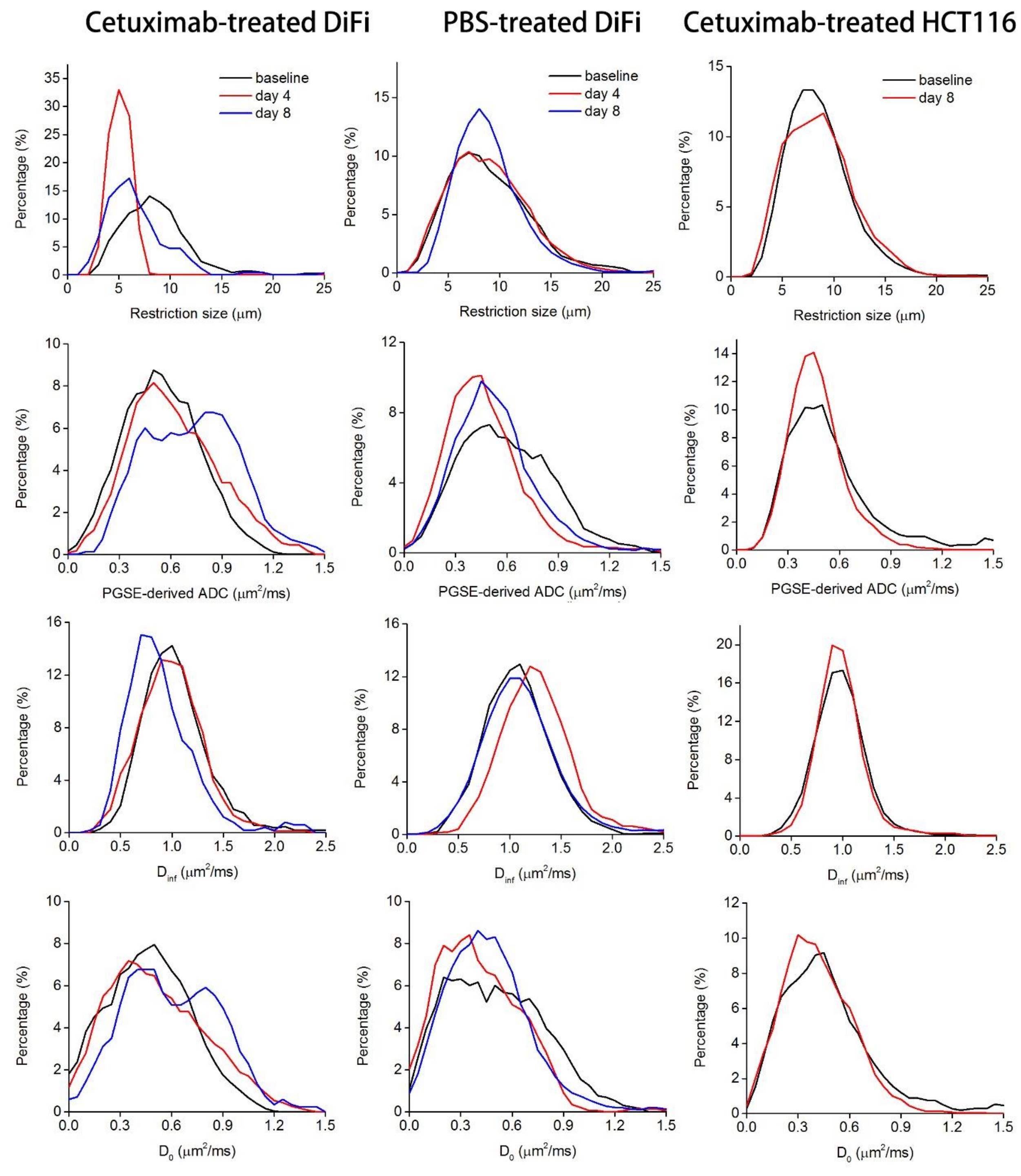

Supplemental Figure 1. Histograms of the PGSE-derived ADC and three TDSI-derived parameters (d, D_{inf}, and D_0) for the same tumor (either cetuximab-treated DiFi, PBS-treated DiFi, or cetuximab-treated HCT116) at day-0/baseline (no treatment), day-4 (2 treatments), and day-8 (4 treatments).
